# Ambulatory Blood Pressure Monitoring in Adults with Congenital Heart Disease

**DOI:** 10.3390/jcm15218359

**Published:** 2026-10-27

**Authors:** Aaron J. Kiel, Priya R. Gupta, Robyn Matloff, Whitney Fairchild, Frank Han, Shailendra Upadhyay, Anudeep K. Dodeja

**Affiliations:** 1 Department of Medicine, Beth Israel Deaconess Medical Center, Harvard Medical School, Boston, MA 02215, USA; 2 School of Medicine, University of Connecticut, Farmington, CT 06030, USA; 3 Department of Pediatrics, Mass General Brigham for Children, Harvard Medical School, Boston, MA 02114, USA; 4 Department of Nephrology, Connecticut Children’s, Hartford, CT 06106, USA; 5 Department of Cardiology, Connecticut Children’s, Hartford, CT 06106, USA; 6 Division of Pediatric Cardiology, Department of Pediatrics, University of Miami Miller School of Medicine, Miami, FL 33136, USA; 7 Holtz Children’s Hospital, Jackson Health System, Miami, FL 33136, USA

**Keywords:** hypertension, ambulatory blood pressure monitor, adult congenital heart disease, white coat hypertension, masked hypertension

## Abstract

**Background/Objectives:** Adults with congenital heart disease (ACHD) are at high risk for late cardiac sequelae and hypertension (HTN). Ambulatory blood pressure monitoring (ABPM) is superior to office measurements in reproducibility, association with cardiovascular outcomes, and detection of masked and white coat HTN (WCH). The utility of ABPM in the ACHD population is not well studied. This study aims to determine the prevalence of hypertension in the ACHD population and evaluate the utility of ABPM in diagnosing hypertension in the ACHD population. **Methods:** This is a single-center retrospective analysis of ACHD patients who completed ABPM from 2021 to 2024. **Results:** Forty ACHD patients underwent ABPM during the study period. Twenty-nine (73%) had hypertension (HTN) in clinic. On ABPM, nine (23%) were confirmed normotensive, 15 (38%) had white coat hypertension (WCH), 14 (35%) were confirmed hypertensive, and two (5%) had masked HTN. There was a 33% decrease in patients meeting criteria for HTN on ABPM compared to in clinic (*p* = 0.002). Ten patients with HTN on ABPM had follow-up, during which seven (70%) had improved clinic blood pressure. One patient (2.5%) had an antihypertensive removed following WCH diagnosis on ABPM. **Conclusions:** ABPM is a useful way to diagnose and monitor hypertension in the ACHD population. Based on ABPM, many patients (43%) were found to have either WCH or masked HTN, and 15% had their HTN stage reclassified. ABPM results led to 33% of patients having subsequent medication adjustment. Data from ABPM can help optimize medical management, in turn reducing cardiac strain and acquired heart disease risk in ACHD patients.

## 1. Introduction

As treatments for congenital heart disease (CHD) have improved, there is an increasing number of adults with CHD (ACHD) [1]. It is estimated that about 85–90% of children born with CHD in Western countries will reach adulthood, such that there are now more adults living with CHD than children [1]. Approximately 2 million adults in the United States are living with CHD, and the number is anticipated to increase at a rate of 5% per year [2,3,4]. As this population grows older, more research is needed on the long-term outcomes these patients experience. Adult congenital heart disease (ACHD) patients are at risk for long-term cardiac sequelae which can be complicated by hypertension (HTN).

The prevalence of hypertension (HTN) in adults with various forms of CHD has been estimated to be between 21 and 47% and has been increasing over recent years [5,6,7,8]. Certain forms of CHD are known to be associated with an increased risk of HTN. For example, about 30% of children with coarctation of the aorta (CoA) will develop HTN, even with early repair [5]. Further, up to 68% of adults living with surgically repaired CoA were found to have HTN [5]. It has been suggested that this increase is, in part, due to increased aortic stiffness and other aortic wall abnormalities [5]. Patients with diagnoses across the ACHD spectrum, including tetralogy of Fallot, transposition of the great vessels, single ventricle, and persistent truncus arteriosus, share these findings, suggesting that patients with these conditions may be at an increased risk of HTN as well [5].

The complex cardiac anatomy of ACHD patients puts them at increased risk of developing cardiac disease, including ventricular hypertrophy, systolic dysfunction, and arrhythmias, all of which can be worsened by untreated HTN [5]. Specifically, untreated HTN in ACHD contributes to the development of ventricular hypertrophy due to increased afterload in an already structurally abnormal heart, and has also been associated with an increased lifetime risk of myocardial infarction, stroke, aortic dissection, cardiac transplantation and death [5]. HTN has also been shown to increase the risk for aortic dilatation in a broad spectrum of ACHD patients, including those with coarctation repair, bicuspid aortic valve, tetralogy of Fallot, and transposition of the great arteries following arterial switch operation [9]. Due to the higher incidence and earlier onset of acquired cardiovascular disease in ACHD patients, the established literature supports earlier and proactive prevention and monitoring of blood pressure across this population, yet there are no currently established guidelines prompting increased blood pressure (BP) screenings or earlier intervention in this population [5,10].

Ambulatory blood pressure monitoring (ABPM) allows for multiple blood pressure measurements over a 24 h period, giving a more accurate and complete picture of the patient’s blood pressure patterns. Since these serial measurements are then averaged, inaccurate measurements or those affected by short-term confounding factors have smaller impacts on the final results. This technique provides many advantages over single measurements at office visits, where blood pressure readings may be confounded by many other variables, such as healthcare anxiety. ABPM is commonly used to determine if a patient’s elevated BP in the office is truly hypertension or “white coat hypertension”, a psychophysiological phenomenon where patients have high BP in a physician’s office but are normotensive outside of the office setting [5]. ABPM may also be used to diagnose “masked hypertension,” where a patient has normal BP in the office but is hypertensive outside the office setting [11]. Finally, since patients are encouraged to follow their normal routine during ABPM, this data can be used to determine what times of day the patient’s BP becomes elevated, which is useful in determining the most effective treatment plan for the individual [11]. In ACHD patients, ABPM has been used to diagnose HTN. One study that used ABPM found that 47% of patients with repaired CoA experienced masked hypertension, suggesting that ABPM would be useful in accurately diagnosing HTN in these patients [11]. Another study in patients with CoA found that 55% of patients were hypertensive on ABPM, 36% of which were newly diagnosed on that ABPM [12]. They also found that only 15% of patients with known HTN had well-controlled BP on ABPM, again showing the utility of ABPM in accurately diagnosing and monitoring HTN [12]. However, there is a gap in the current evidence regarding the prevalence of hypertension in the ACHD population as a whole, as well as a lack of evidence for the utility of ABPMs to measure blood pressure in this population. This study aims to contribute to the body of scientific knowledge in this area.

## 2. Materials and Methods

### 2.1. Study Population

This study was granted an exemption from the Connecticut Children’s IRB. This is a single-center retrospective study performed at the Adult Congenital Heart Disease Program at Connecticut Children’s in Hartford, CT. Patients were included if they met the following criteria: (1) patient of the Adult Congenital Heart Disease Program at Connecticut Children’s, (2) age 18 years old or older, (3) diagnosed with a CHD listed in Table A1, and (4) completed 24 h ambulatory blood pressure monitoring between January 2021 and December 2024. Patients who had completed ABPM within the study time period were identified by a search of the electronic medical record. Patients were then excluded for the following reasons: (1) no CHD diagnosis, (2) age under 18 years old, and (3) incomplete data on ABPM. CHD complexity was classified using the AHA ACHD Anatomy classification system (Table A1) [13]. Patients were classified as simple, moderate, or complex based on their CHD diagnosis. If patients had multiple CHD diagnoses in different categories, they were placed in the highest of the categories.

### 2.2. Study Outcomes

The primary outcome variable was blood pressure, as measured in clinic or by an ambulatory blood pressure monitor (Table 1). The proportion of the population found to have hypertension in clinic or on ABPM was determined using a modified version of the 2017 ACC/AHA guidelines (Table 1) [14]. Some ABPM reports from this timeframe at our institution used 2008 AHA criteria to classify patients using systolic and diastolic load [15]. These reports were manually reclassified using the blood pressure categories in Table 1. ABPM diagnosis was determined based on 24 h mean. Nocturnal hypertension was defined as stage 1 or 2 HTN on ABPM without meeting criteria for HTN based on 24 h ABPM.

White coat hypertension (WCH) was diagnosed if the patient met criteria for hypertension in the office but did not meet hypertension criteria on ABPM. Masked hypertension was diagnosed if the patient did not meet criteria for hypertension in office but did meet criteria for HTN on ABPM. Isolated diastolic HTN was defined as diastolic BP meeting criteria for HTN without systolic HTN. The number of patients with hypertension in clinic was compared to the number with hypertension on ABPM using McNemar’s exact and asymptotic testing. Results of ABPM were also stratified by complexity of CHD and by CoA status. Fisher’s exact testing was used to compare the proportion of ABPM-diagnosed HTN in patients with and without CoA. Fisher’s exact testing was also used to compare HTN prevalence by CHD complexity.

## 3. Results

### 3.1. Population Demographics

**Figure 1:**
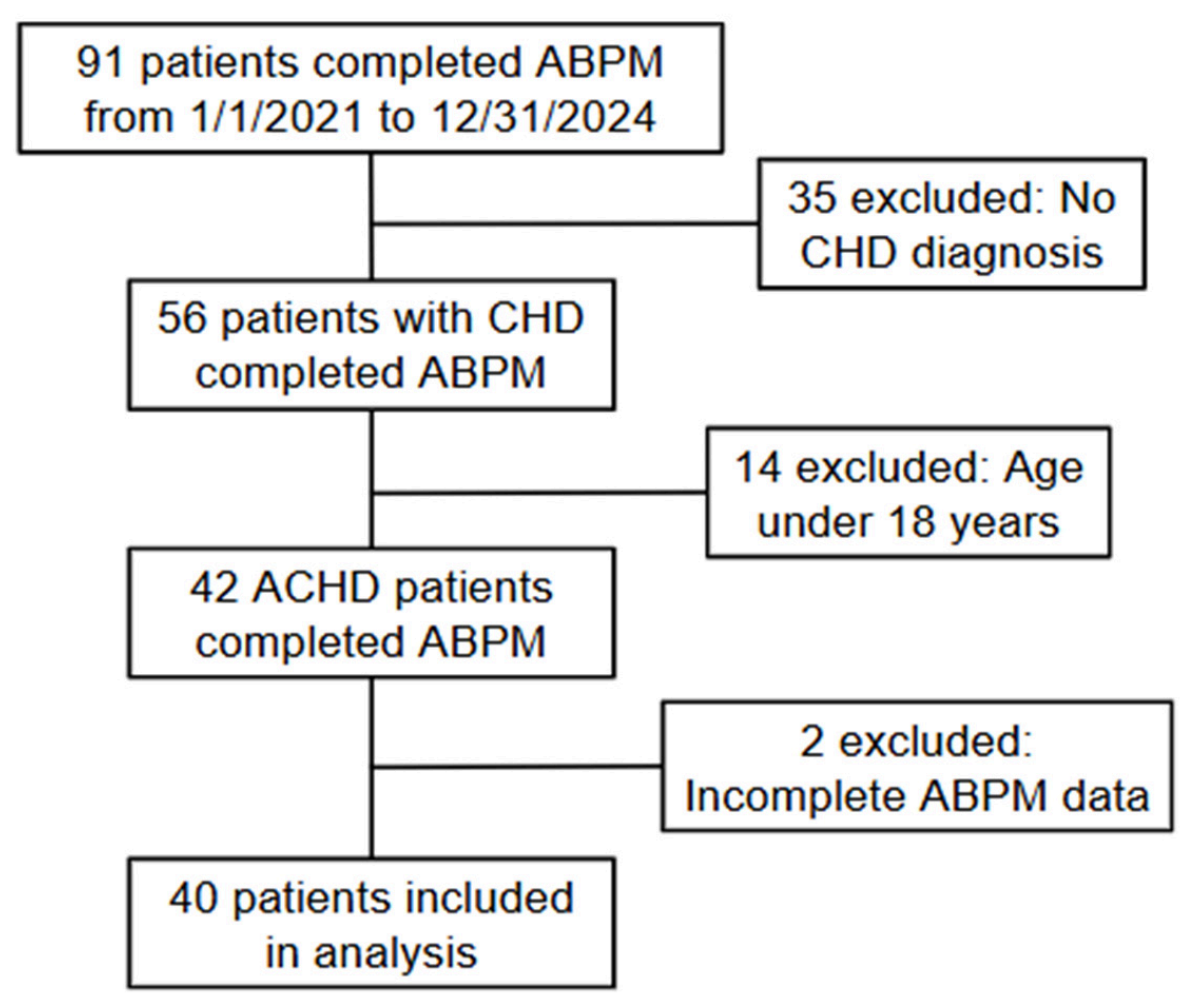
Development of study cohort. Patients who had no CHD diagnosis, were under the age of 18, or had incomplete ABPM data were excluded from the study, resulting in a study population of 40 patients. Abbreviations: ambulatory blood pressure monitor (ABPM); congenital heart disease (CHD).

Ninety-one patients completed ABPM from January 2021 to December 2024, of which 40 met the inclusion criteria for this study (Figure 1). Patient characteristics are shown in Table 2. The most common CHD diagnoses were CoA (38%), bicuspid aortic valve (33%), VSD (20%), variations of single ventricle (15%), tetralogy of Fallot (10%), mitral valve disease (10%), and PDA (8%), with some patients having multiple diagnoses. Indications for ordering the ABPM included an elevated BP reading in clinic (23%), CoA (18%), essential HTN (13%), secondary HTN (10%), and ACHD patients known to be at risk for HTN, with some patients having multiple indications.

### 3.2. Hypertension

**Figure 2:**
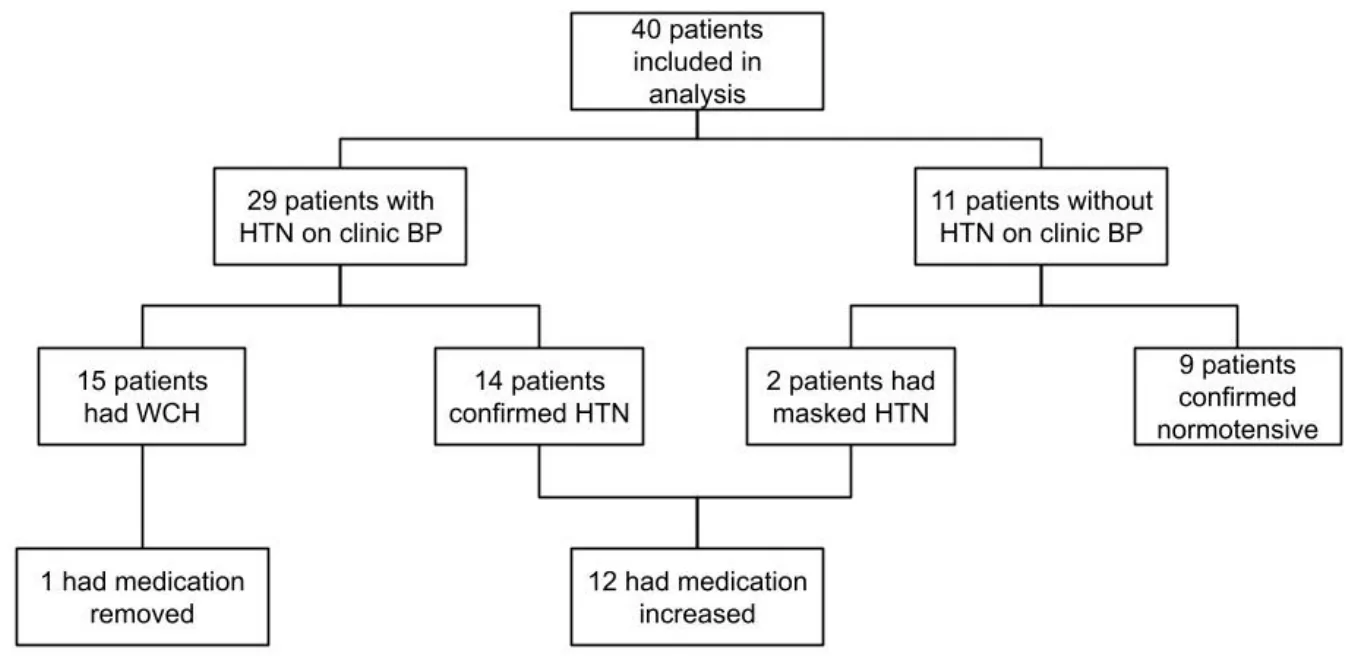
Results of initial ambulatory blood pressure monitoring. Out of the 40 patients in this study, 29 had HTN in office. Of these, 15 were found to have WCH and 14 had true HTN. Of the 11 patients without HTN in office, two were found to have masked HTN and nine were truly normotensive. Abbreviations: blood pressure (BP); hypertension (HTN); white coat hypertension (WCH).

The majority of patients, 29 (73%), had HTN in clinic, with mean BP 132 ± 14/80 ± 12 mmHg (Figure 2). Of these, 12 (30%) had stage 1 HTN and 17 (43%) had stage 2 HTN. Nine (23%) were normotensive in clinic and two (5%) had elevated BP in clinic. On ABPM, 16 (40%) had HTN, of which four (25%) had stage 1 and 12 (75%) had stage 2. Mean daytime BP was 124 ± 15/73 ± 10, mean nighttime BP was 109 ± 14/62 ± 10, and mean 24 h BP was 118 ± 14/70 ± 10. Eleven (28%) were normotensive, and 13 (33%) had elevated BP. Based on the ABPM results, 17 patients (43%) were reclassified either as WCH (15) or as masked HTN (2). Both of the patients with masked HTN had stage 2 HTN. Fourteen of the 29 patients with HTN in clinic (48%) were confirmed to have HTN. Four patients with stage 2 HTN in clinic were downgraded to stage 1 HTN, two patients with stage 1 HTN in clinic were upgraded to stage 2 HTN, and eight patients had their clinic HTN stage confirmed. Eleven (28%) patients had nocturnal hypertension only on ABPM. Two (5%) patients had isolated diastolic HTN in clinic and were confirmed to have isolated diastolic HTN based on 24 h mean ABPM. McNemar’s test comparing HTN in clinic to HTN on ABPM found a 33% decrease (asymptotic 95% CI 13% to 48%) in the number of patients with HTN on ABPM compared to the number of patients with HTN in clinic, with exact *p* = 0.002 (Table 3). Fisher’s exact testing comparing HTN prevalence (confirmed HTN and masked HTN) in patients with and without CoA gave *p* = 1.000 (Table 4). Fisher–Freeman–Halton testing comparing HTN prevalence (confirmed HTN and masked HTN) by CHD complexity gave *p* = 0.220 (Table 4).

### 3.3. Medication Management

Prior to completion of ABPM, nine (23%) patients received no treatment for blood pressure, five (13%) patients were advised lifestyle changes with diet and exercise, and twenty-six patients were on antihypertensive medication, including 16 (40%) patients who were treated with one antihypertensive and 10 (25%) patients who were prescribed more than one antihypertensive. Seventeen (42.5%) patients were medicated with an angiotensin-converting-enzyme inhibitor (ACEi) or angiotensin receptor blocker (ARB), 12 (30%) were on a beta blocker, eight (20%) were on a diuretic antihypertensive, and six (15%) were on a calcium channel blocker. Of the twenty-six patients on antihypertensive therapy, seven (18%) patients did not have any history of hypertension. Other indications for antihypertensives included congestive heart failure (five), stroke (three), arrhythmias (three), aortic root dilation (two), left ventricular dysfunction (one), idiopathic intracranial HTN (one), and hypokalemia (one). Of the 19 patients being treated for HTN prior to ABPM, nine had well-controlled BP on ABPM. Among those with HTN on ABPM, 12 (63%) had subsequent medication adjustment, including nine (23%) who had an antihypertensive medication added and three (8%) who had the dose of their existing antihypertensive increased. After medication adjustment, 10 (83%) had follow-up clinic visits and three (25%) had follow-up ABPM. During follow-up visits, seven (70%) had improved clinic BP compared to before the ABPM. On follow-up ABPM, one (33%) demonstrated improved HTN control. One patient (2.5%) had an antihypertensive removed following WCH diagnosis and low nocturnal blood pressure on ABPM in combination with symptoms of lightheadedness. At a follow-up clinical visit, their blood pressure was relatively unchanged but their lightheadedness had resolved.

### 3.4. Follow-Up ABPM

Overall, seven (18%) patients had repeat ABPM, six (86%) of whom had been diagnosed with HTN on their first monitor. Of the six who had HTN on initial ABPM, one (17%) was normotensive on follow-up ABPM, one (17%) had improvement but still elevated BP, three (50%) had persistent stage 1 HTN, and one (17%) had HTN that had worsened from stage 1 to stage 2. The two patients with persistent stage 1 HTN and the patient who worsened from stage 1 to stage 2 had additional follow-up ABPMs, eventually becoming normotensive. The patient who was normotensive on initial ABPM had their dose of antihypertensive decreased after the first ABPM, leading to follow-up with a second ABPM. On follow-up ABPM after this medication change, they were found to have developed stage 1 HTN. After this second ABPM showed stage 1 HTN, an additional antihypertensive was added and a third ABPM was done, which was normotensive.

## 4. Discussion

### 4.1. Hypertension Diagnosis

Results from this study demonstrate the utility of using ABPM to confirm high blood pressure diagnoses in the ACHD population. When ABPM was used to diagnose HTN, a lower proportion of the population met the criteria, indicating that some elevated BP readings in clinic reflect WCH rather than true HTN. Our study also found a decrease in the number of patients with HTN on ABPM, with a statistically significant 33% decrease (*p* = 0.002) compared to in-clinic HTN, demonstrating the utility of ABPM in accurately diagnosing HTN.

Using only clinic-based measurements for these patients may have led to over-treatment with antihypertensives. On the other hand, there were multiple patients that were normotensive in the clinic setting, but diagnosed with HTN by ABPM. These patients would have likely gone undiagnosed and untreated if they had not received ABPM. Between the WCH and masked HTN patients, 17 (43%) of the patients in the study had their diagnosis changed based on ABPM results. Additionally, almost half (43%) of the patients who had HTN both in clinic and on ABPM had their stage of HTN reclassified, with most of them switching from stage 2 HTN to stage 1 HTN. This suggests that ABPM may also help to more accurately stratify a patient’s need for HTN treatment. In this study, nearly one-third of all patients had nocturnal HTN without daytime HTN, which may require different screening or treatment plans. Finally, ABPM can be used to identify if a patient has HTN or only isolated diastolic HTN, which may affect their treatment plan.

### 4.2. Previous Studies

Overall, our results are consistent with other studies, which have studied ABPM in patients with CoA and found that ambulatory blood pressure correlates with end-organ damage better than clinic BP, suggesting that ABPM is the most accurate way to assess BP in the CoA population [16]. Our study is novel in that we include patients with all ACHD diagnoses, rather than only coarctation. To our knowledge, there have not been previous studies comparing clinic BP to ABPM results across the spectrum of ACHD. Previous studies found that between 30–47% of participants with CoA had masked hypertension [11,16]. Our study found that one out of the 15 patients with CoA and two patients overall had masked hypertension, again highlighting the importance of screening ACHD individuals for HTN with ABPM. While this study was underpowered to detect a true difference between individuals with and without CoA, our data suggests all ACHD patients would benefit from screening with ABPM.

Another study performed ABPM for CoA patients who had a diagnosis of hypertension, and found that hypertension was well controlled in only 30% of the group [11]. Our study also found that only a minority of patients with known hypertension had good BP control on ABPM. In our study, the majority of patients with hypertension on ABPM had medication adjustments based on ABPM results, leading to improved clinic BP at the follow-up visit. One patient with WCH was taken off of their antihypertensive due to ABPM findings, with resolution of symptoms that were attributed to medication side effects.

### 4.3. Utility of ABPM

This data demonstrates that ABPM is a valuable tool for all ACHD patients. ABPM can be used to diagnose and stage HTN, and can be used to make meaningful changes to medication regimens. ABPM has few disadvantages, with the most important being the availability and cost of the device, discomfort during measurement, and patient compliance [4]. Given the large proportion of these ACHD patients that had their clinic diagnosis changed based on ABPM data, the benefits likely outweigh the risks. Thus, ABPM should be used to screen for HTN if there is strong suspicion of or risk factors for HTN and to confirm the diagnosis of HTN prior to treatment.

### 4.4. Study Limitations

This is a single-center retrospective study with a small sample size due to the overall low prevalence of congenital heart disease. Our sample size does limit generalizability, and as such, findings from the study should be interpreted as descriptive and hypothesis-generating. This study can help identify patterns that can inform the design of larger multicenter studies. In this study, patients’ ABPM diagnosis was manually determined using a modified version of the 2017 ACC/AHA criteria. However, upon review of ABPM, some reports used 2008 AHA criteria to classify patients using systolic and diastolic load [15]. This may have guided providers’ decisions to treat or not treat patients differently than would be done based on the current criteria, affecting which patients had medication adjustments. Additionally, patients in this study were offered ABPM due to suspected or diagnosed hypertension. This may have affected the results as this population may have been more likely to have hypertension on ABPM than patients without clinical suspicion for hypertension. Despite this, nine of the patients in this study were normotensive in clinic, of which two were found to have hypertension on ABPM. This data does suggest that ABPM can be useful in diagnosing or ruling out hypertension when it is suspected, but these findings should be confirmed in a larger population of patients without known hypertension. Finally, the authors acknowledge that the study group was heterogenous, including ACHD patients with all forms of congenital heart disease and varying disease severity. Our limited sample size did not allow for stratification of results by individual CHD diagnoses. However, looking at the prevalence of HTN in the ACHD population as a whole remains important because despite anatomic heterogeneity, patients share lifelong alterations in anatomy, prior surgical exposure, vascular abnormalities, and the potential for other acquired cardiovascular risk factors [5].

## 5. Conclusions

Ambulatory blood pressure monitoring is a valuable tool that has been understudied and likely underutilized in ACHD patients. ABPM can be used to confirm suspected hypertension, assess hypertension control, identify white coat hypertension, and reveal masked hypertension. Our study found a statistically significant decrease in patients diagnosed with HTN on ABPM compared to clinic BP measurement. Based on ABPM, a significant proportion of our patients (43%) were reclassified either as WCH or masked HTN, and 15% had their HTN stage adjusted. Further, ABPM results led to 33% of patients having subsequent medication adjustment. Data from ABPM can facilitate optimization of medical management, in turn reducing cardiac strain and acquired heart disease risk in patients who have vulnerable myocardial health. While data from this study is supportive of ambulatory blood pressure monitoring as a successful method for identifying forms of hypertension in ACHD patients, larger multicenter studies with longer follow-up are needed for confirmation and to further elucidate differences in HTN based on CHD complexity.

## Figures and Tables

**Table 1:** Blood pressure categories based on the 2017 ACC/AHA guidelines [14].

BP Category	Clinic (mmHg)	Daytime ABPM (mmHg)	Nighttime ABPM (mmHg)	24 h ABPM (mmHg)
Normal	SBP < 120 and DBP < 80	SBP < 120 and DBP < 80	SBP < 100 and DBP < 65	SBP < 115 and DBP < 75
Elevated	SBP 120–129 and DBP < 80	SBP 120–129 and DBP < 80	SBP 100–109 and DBP < 65	SBP 115–124 and DBP < 75
Stage 1 HTN	SBP 130–139 or DBP 80–89	SBP 130–134 or DBP 80–84	SBP 110–119 or DBP 65–69	SBP 125–129 or DBP 75–79
Stage 2 HTN	SBP ≥ 140 or DBP ≥ 90	SBP ≥ 135 or DBP ≥ 85	SBP ≥ 120 or DBP ≥ 70	SBP ≥ 130 or DBP ≥ 80
Isolated diastolic HTN	SBP < 130 and DBP ≥ 80	SBP < 135 and DBP ≥ 85	SBP < 120 and DBP ≥ 70	SBP < 130 and DBP ≥ 80

Abbreviations: ABPM, ambulatory blood pressure monitor; BP, blood pressure; DBP, diastolic blood pressure; HTN, hypertension; mmHg, millimeters of mercury; SBP, systolic blood pressure.

**Table 2:** Demographic information and medical history of this patient cohort.

Patient Characteristic	Frequency, n (%)
Gender	
Male	19 (48)
Female	21 (53)
Age (mean: 32 ± 13 years old)	
18–39 years old	30 (75)
40–64 years old	7 (18)
65+ years old	3 (8)
Comorbidities	
Overweight (24.9 < BMI < 29.9)	10 (25)
Obesity (BMI > 29.9)	15 (38)
Diabetes	3 (8)
Hyperlipidemia	12 (30)
Tobacco smoker	5 (13)
Turner’s syndrome	2 (5)
CHD complexity	
Simple	3 (8)
Moderate	28 (70)
Complex	9 (23)

Abbreviations: BMI, body mass index; CHD, congenital heart disease.

**Table 3:** Results of McNemar’s testing.

	ABPM: HTN	ABPM: No HTN	Total
Clinic: HTN	14	15	29
Clinic: No HTN	2	9	11
Total	16	24	40

Abbreviations: ABPM, ambulatory blood pressure monitoring; HTN, hypertension.

**Table 4:** Results of ABPM stratified by CoA status and CHD complexity.

	CoA Status		CHD Complexity		
Diagnosis	CoA, n (%)	No CoA, n (%)	Simple, n (%)	Moderate, n (%)	Complex, n (%)
Confirmed HTN	5 (33)	9 (36)	0 (0)	8 (30)	6 (67)
WCH	6 (40)	9 (36)	1 (33)	12 (44)	2 (22)
Masked HTN	1 (7)	1 (4)	0 (0)	2 (7)	0 (0)
Confirmed Normal BP	3 (20)	6 (24)	2 (67)	6 (22)	1 (11)

Abbreviations: ABPM, ambulatory blood pressure monitor; BP, blood pressure; CHD, congenital heart disease; CoA, coarctation of aorta; HTN, hypertension; WCH, white coat hypertension.

## Data Availability

The original contributions presented in this study are included in the article. Further inquiries can be directed to the corresponding authors.

## Appendix A

**Table A1:** List and classification of congenital heart diseases [13].

CHD Anatomy
**Class I: Simple**
Isolated small ASD
Isolated small VSD
Mild isolated pulmonic stenosis
Previously ligated or occluded ductus arteriosus
Repaired secundum ASD or sinus venosus defect without significant residual shunt or chamber enlargement
Repaired VSD without significant residual shunt or chamber enlargement
**Class II: Moderate**
Aorto-left ventricular fistula
Anomalous pulmonary venous connection, partial or total
Anomalous coronary artery arising from the pulmonary artery
Anomalous aortic origin of a coronary artery from the opposite sinus
AVSD
Congenital aortic valve disease
Congenital mitral valve disease
Coarctation of the aorta
Ebstein anomaly
Infundibular right ventricular outflow obstruction
Ostium primum ASD
Moderate and large unrepaired secundum ASD
Moderate and large persistently patent ductus arteriosus
Pulmonary valve regurgitation, moderate or greater
Pulmonary valve stenosis, moderate or greater
Peripheral pulmonary stenosis
Sinus of Valsalva fistula/aneurysm
Sinus venosus defect
Subvalvar aortic stenosis (excluding HCM)
Supravalvar aortic stenosis
Straddling atrioventricular valve
Repaired tetralogy of Fallot
VSD with associated abnormality and/or moderate or greater shunt
**Class III: Complex**
Cyanotic congenital heart disease
Double outlet ventricle
Fontan procedure
Interrupted aortic arch
Mitral atresia
Single ventricle (double inlet left ventricle, tricuspid atresia, hypoplastic left heart, any other anatomic abnormality with functional single ventricle)
Pulmonary atresia
TGA (classic or d-TGA)
Truncus arteriosus
Other abnormalities of atrioventricular and ventriculoarterial connection (crisscross heart, isomerism, heterotaxy syndromes, ventricular inversion)

Abbreviations: ASD, atrial septal defect; AVSD, atrioventricular septal defect; HCM, hypertrophic cardiomyopathy; TGA, transposition of the great arteries; VSD, ventricular septal defect.

## References

[1] Gurvitz M., Burns K.M., Brindis R., Broberg C.S., Daniels C.J., Fuller S.M.P.N., Honein M.A., Khairy P., Kuehl K.S., Landzberg M.J. (2016). Emerging Research Directions in Adult Congenital Heart Disease: A Report From an NHLBI/ACHA Working Group. J. Am. Coll. Cardiol..

[2] Nguyen L.T., Maul T.M., Hindes M., Daniels C.J., Gurvitz M., Cook S.C. (2015). Current and future status of adult congenital training in North America. Am. J. Cardiol..

[3] Warnes C.A. (2005). The adult with congenital heart disease: Born to be bad?. J. Am. Coll. Cardiol..

[4] Shekhar S., Agrawal A., Pampori A., Lak H., Windsor J., Ramakrishna H. (2022). Mortality in Adult Congenital Heart Disease: Analysis of Outcomes and Risk Stratification. J. Cardiothorac. Vasc. Anesth..

[5] Sabatino J., Avesani M., Sirico D., Reffo E., Castaldi B., Bassareo P., Di Salvo G. (2023). Systemic hypertension in adults with congenital heart diseases. Int. J. Cardiol. Congenit. Heart Dis..

[6] Raskind-Hood C., Gray K.A., Morgan J., Book W.M. (2021). Hypertension and heart failure as predictors of mortality in an adult congenital heart defect population. Congenit. Heart Dis..

[7] Pieles G., Papadakis M., Budts W. (2023). Exercise, prescription and training in ACHD. Int. J. Cardiol. Congenit. Heart Dis..

[8] Agarwal S., Sud K., Khera S., Kolte D., Fonarow G.C., Panza J.A., Menon V. (2016). Trends in the Burden of Adult Congenital Heart Disease in US Emergency Departments. Clin. Cardiol..

[9] Lui G.K., Fernandes S., McElhinney D.B. (2014). Management of cardiovascular risk factors in adults with congenital heart disease. J. Am. Heart Assoc..

[10] Borrelli N., Sabatino J., Ruperti-Repilado F.J., Tutarel O., Giannakoulas G., D’Alto M., Romeo E., Tobler D., Diller G.P. (2025). New perspectives in the management of adults with congenital heart disease: Insights from the 2025 ACHD Congress. Eur. Heart J. Suppl..

[11] Di Salvo G., Castaldi B., Baldini L., Gala S., del Gaizo F., D’Andrea A., Limongelli G., D’Aiello A.F., Scognamiglio G., Sarubbi B. (2011). Masked hypertension in young patients after successful aortic coarctation repair: Impact on left ventricular geometry and function. J. Hum. Hypertens..

[12] Fedchenko M., Mandalenakis Z., Dellborg H., Hultsberg-Olsson G., Björk A., Eriksson P., Dellborg M. (2019). Cardiovascular risk factors in adults with coarctation of the aorta. Congenit. Heart Dis..

[13] American Heart Association The 2025 Adult Congenital Heart Disease (ACHD) Anatomic and Physiological Classification System. https://professional.heart.org/en/science-news/2025-adult-congenital-heart-disease-anatomic-and-physiological-classification-system.

[14] Whelton P.K., Carey R.M., Aronow W.S., Casey D.E., Collins K.J., Himmelfarb C.D., DePalma S.M., Gidding S., Jamerson K.A., Jones D.W. (2018). 2017 ACC/AHA/AAPA/ABC/ACPM/AGS/APhA/ASH/ASPC/NMA/PCNA Guideline for the Prevention, Detection, Evaluation, and Management of High Blood Pressure in Adults: Executive Summary: A Report of the American College of Cardiology/American Heart Association Task Force on Clinical Practice Guidelines. Hypertension.

[15] Urbina E., Alpert B., Flynn J., Hayman L., Harshfield G.A., Jacobson M., Mahoney L., McCrindle B., Mietus-Snyder M., Steinberger J. (2008). Ambulatory blood pressure monitoring in children and Adolescents: Recommendations for standard assessment. Hypertension.

[16] Egbe A., Banala K., Vojjini R. (2020). Potential benefits of ambulatory blood pressure monitoring in coarctation of aorta. Eur. Heart J..

